# Early Age Sealing Capacity of Structural Mortar with Integral Crystalline Waterproofing Admixture

**DOI:** 10.3390/ma14174951

**Published:** 2021-08-30

**Authors:** Calin Mircea, Tudor-Panfil Toader, Andreea Hegyi, Brăduț-Alexandru Ionescu, Andreea Mircea

**Affiliations:** 1 Civil Engineering Faculty, Technical University of Cluj-Napoca, 28 Memorandumului Street, 400114 Cluj-Napoca, Romania; tudor.toader@incerc-cluj.ro (T.-P.T.); bradut.ionescu@incerc-cluj.ro (B.-A.I.); andreea.mircea@ccm.utcluj.ro (A.M.); 2 NIRD URBAN-INCERC Cluj-Napoca Branch, 117 Calea Floressti, 400524 Cluj-Napoca, Romania; andreea.hegyi@incerc-cluj.ro

**Keywords:** mortar, self-sealing, micro-cracks, crystalline admixtures, early age, grain size, probabilistic analysis

## Abstract

Crystalline admixtures embedded in concrete may react in the presence of water and generate thin crystals able to fill pores, capillaries and micro-cracks. Once the concrete has dried, the crystalline chemicals sit dormant until another dose of water starts the crystallization again. The research aims to analyses the early age self-sealing effect of a crystalline admixture at a dosage rate of 1–3% of the cement mass. Specimens made with two types of gravel were pre-loaded with three-point bending to up to 90% of the ultimate capacity, and conditioned through wet–dry cycles. Micro-crack closure was measured with a microscope after pre-loading, and after 1 day, 4 days, 8 days, 14 days and 20 days of wet–dry exposure. The results show that an admixture content of 3% achieves the best early self-sealing performance. These results are also confirmed by probabilistic analyses, which also emphasize the self-sealing potential of lower ICW contents.

## 1. Introduction

Cementitious composites have been widely used all over the world since the spectacular development of Portland Cement in the 19th century. Even in traditional/initial forms (e.g., ordinary concrete), cementitious composites have proven their remarkable autogenous self-healing ability, as became well known in the same period [1,2], attributed at that time to the ongoing hydration reaction. However, the phenomenon was neglected for many decades, and it was not until the end of the 19th century when researchers’ interest was piqued by this self-sealing potential and its ability to reduce early age cracking [3,4,5]. More recently, the contribution of tensile creep was also considered for restrained shrinkage, and significant research has been initiated on the topic to quantify the creep tensile strain [6,7,8].

Self-healing can potentially have a great impact upon the durability of concrete structures, especially on those exposed to severe conditions. Within the last few decades, significant research has been done to improve the knowledge of the relevant mechanisms, and to develop new concepts and techniques [9,10,11,12,13,14,15,16]. We can now identify three major self-healing processes/techniques: autogenous self-healing, encapsulated autonomous self-healing and self-healing bio-concrete. Given the nature of the present work, only autogenous self-healing will be discussed in this paper.

Cementitious materials possess a natural autogenous healing capacity by filling micro-cracks through four mechanisms: the continued hydration of unhydrated cement, calcium carbonate precipitation, the expansion/swelling of calcium-silicate hydrate gel, and the deposition of impurities such as water (e.g., debris and spall crack) [17,18,19,20]. The first two mechanisms are the most important. At an early age, continued hydration seems to make the greatest contribution, while for mature concrete the second mechanism becomes essential [21]. Crack widths of up to 300–400 μm have been reported to be cured by autogenous healing in the presence of water [12,22]. The major factors that influence the process are mix composition, concrete age, water presence and crack shape and width [23,24]. Besides the natural/intrinsic ability, autogenous healing potential can also be stimulated. Mineral additives, such as blast-furnace slag and fly ash, significantly improve autogenous healing when used to partially replace Portland cement [18,25,26,27,28,29,30] through ongoing hydration. Limestone powder, marble slurry and hydrated lime increase the calcium content and act as a site for the nucleation of cement hydration [31,32,33,34], being sustainable replacements for cement [35]. Superabsorbent polymers induce self-healing by providing an ongoing reservoir of water for hydration. By encouraging multiple cracking, more narrow cracks occur [36]. These also stimulate further hydration [37,38]. 

Integral crystalline waterproofing (ICW) admixtures embedded in concrete contain crystalline chemicals that react in the presence of water and generate thin crystals able to fill pores, capillaries and micro-cracks [39]. As long as moisture remains present, crystals continue to grow inside concrete. Once the concrete has dried, the crystalline chemicals sit dormant until another dose of water, e.g., supplied through a new crack, causes the chemical reaction, known as crystallization, to start again [40,41,42,43,44,45]. Mortars with crystalline and expansive additives have also been proven to have higher pH values, which favor calcium carbonate precipitation [22].

The research provides experimental evidence of the early self-sealing potential of ICW admixtures. A powder ICW admixture was added in percentages of 1–3% of the mass of the Portland cement, based on the producer’s recommendations. 

The ICW’s self-sealing potential was assessed on two types of mortar mixes. The first used typical quartz sand with a maximum grain size of 2 mm, in accordance with EN 933-1:2012. The second considered a quartz gravel of up to 4 mm, in an attempt to study the self-sealing potential of the local available gravel in mixes with higher porosity and diversified fracture behavior [46,47,48].

In summary, the main objective of this research was to analyze the early self-sealing potential of mortars with ICW admixtures with a content of 1–3% of the cement mass. A secondary objective was to determine the changes caused by doubling the maximum grain size of the quartz gravel, from 2 mm to 4 mm, giving rise to fracture processes with diverse parameters. The analyses were performed based on tests, wet–dry conditioning, and analyses of self-sealing parameters from a probabilistic perspective.

## 2. Materials and Methods

### 2.1. Ingredients and Mix Proportions

**Figure 1:**
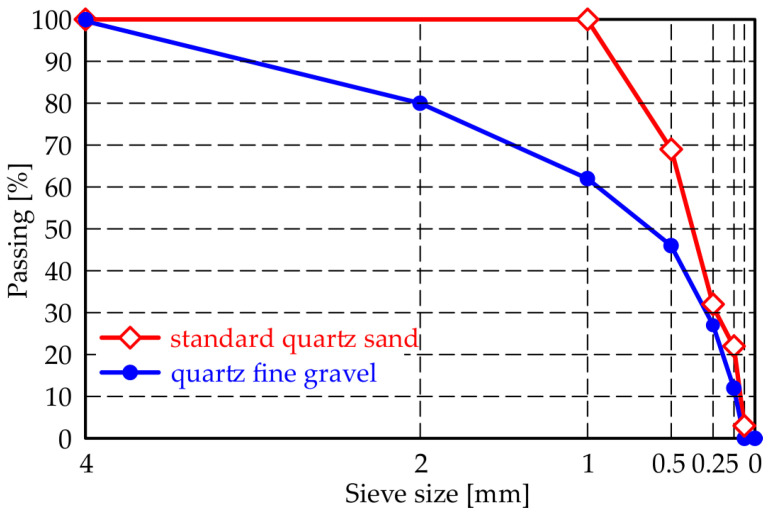
Particle size distribution of the sand and gravel employed in the tested specimens.

The ingredients and proportions were chosen, starting with the reference mix T0r shown in Table 1, which demonstrated a significant self-healing ability in a previous work [49]. For comparison, the same T0c mix was made with quartz gravel instead of standard quartz sand. Both aggregates had a similar chemical composition, with quartz crystals (i.e., SiO_2_) present at more than 96%. Comparative particle size distributions are shown in Figure 1.

A type-C fly ash was added to the Portland cement type CEM I 42.5R, referenced by EN 197-1, in a mass proportion of 1.12. Marble slurry (MS), an important by-product of marble manufacturing, was introduced as a fine aggregate with a size ranging from 75 μm to 4.75 mm. A polycarboxylate-based high-range water-reducing admixture (HRWRA) and synthetic polyvinyl alcohol (PVA) fibers were added to promote the precipitation of crystallization products on the crack’s surface [50,51]. The PVA fibers, with a density of 1300 kg/m^3^, were 8 mm in length and 39 µm in diameter, with a tensile strength of 1.2 GPa and a 1.2% mineral oil coating to reduce the interfacial chemical bond with the matrix. A constant volume fraction of 2% was introduced. 

**Figure 2:**
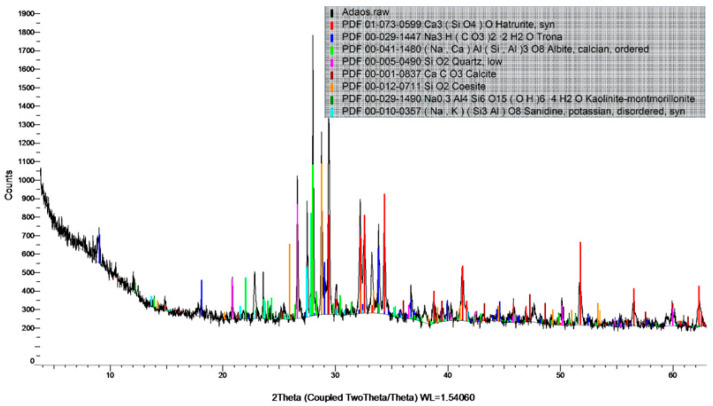
X-ray diffraction result of the ICW powder.

The addition of the ICW powder, with a density of 1.0 g/cm^3^, into the studied mixes was carried out by reducing the C fly ash mass proportion by the same quantity. Figure 2 presents the diffraction pattern of the ICW white powder used. This reveals the high content of hatrurite, trona, calcite, coesite and albite crystals, all with direct impacts upon the self-sealing ability.

The mix proportions are shown in Table 2 in relative mass proportions.

### 2.2. Specimen Preparation, Preloading and Conditioning

**Figure 3:**
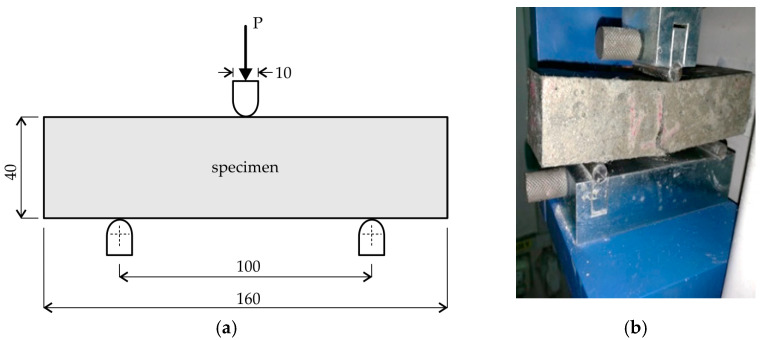
Schematics of the preliminary test set up: (**a**) EN 196-1 three-point bending (dimensions in mm); (**b**) specimen under flexural failure.

After mixing, the fresh mortar mixtures were cast into 40 × 40 × 160 mm molds, covered in plastic foil, and stored in a climatic chamber at 20 ± 2 °C and 90% relative humidity (RH). After 24 h, the specimens were removed from the molds and water-cured for 28 days under room conditions (i.e., 20 ± 2 °C and RH = 50%). After that, three specimens of each mixture were subjected to preliminary three-point bending tests (see Figure 3), and another three specimens were cracked by pre-loading up to 90% of the mean flexural strength (i.e., modulus of rupture) previously determined. The results of the preliminary three-point bending tests are given in Table 3. The densities of the hardened mortar samples, determined with the same standard deviation of ±1%, are also shown in Table 3.

In the end, daily wet–dry cycles were applied for 20 days. Every day, 16 h of water immersion was followed by 8 h of dry exposure under the above room conditions (i.e., 20 ± 2 °C and RH = 50%). 

### 2.3. Self-Sealing Parameters and Evaluation

The micro-crack widths were measured with a Leica DMC2900 microscope just after preloading, and after 1 day, 4 days, 8 days, 14 days and 20 days of wet–dry exposure in daily cycles. Readings were made on segments framed in small areas of 1.6 × 1.3 mm with a magnification factor of 8×. The following time-dependent self-sealing parameters are discussed with regard to both the raw segment data and individual cracks:

- Crack closure, considering the relative decrease in the average crack width w_av_ at day t of conditioning, related to the initial moment after preloading

(1) $$\mathrm{Crack}\;\mathrm{closure}\;[\%]=\frac{w_{\mathrm{av}}\left(0\right)-w_{\mathrm{av}}\left(t\right)}{w_{\mathrm{av}}\left(0\right)}\times 100$$

- Closure rate of the crack, considering the decrease in the average crack width related to the corresponding interval t in days

(2) $$\mathrm{Closure}\;\mathrm{rate}\;[\mu m/\mathrm{day}]=\frac{w_{\mathrm{av}}\left(0\right){-w}_{\mathrm{av}}\left(t\right)}{t}.$$

The choice of the above parameters is justified by the fact that they offer a global characterization of the phenomena. Similar to in [41,52], the average crack width was determined on crack segments w^i^_av_, and full cracks w_av_ were determined by:

(3) $$w_{\mathrm{aw}}^{i}=\frac{A_{\mathrm{cr}}^{i}}{l_{\mathrm{cr}}^{i}}$$

(4) $$w_{\mathrm{av}}=\frac{\sum_{i}A_{\mathrm{cr}}^{i}}{\sum_{i}l_{\mathrm{cr}}^{i}}$$

where A^i^_cr_ is the area of the crack segment i and l^i^_cr_ is the midline crack segment.

The parameters were then analyzed and discussed in the context of the Gaussian distribution, implementing the general form of the probability density function—

(5) $$f\left(x\right)=\frac{1}{\sigma \sqrt{2\pi }}\exp\left[-\frac{1}{2}\frac{{\left(x-\mu \right)}^{2}}{\sigma^{2}}\right]$$

with the variable x (i.e., crack closure and closure rate), µ as the mean value of the variable x, and σ as its standard deviation.

## 3. Results and Discussion

### 3.1. Self-Sealing Crack Closure

**Figure 4:**
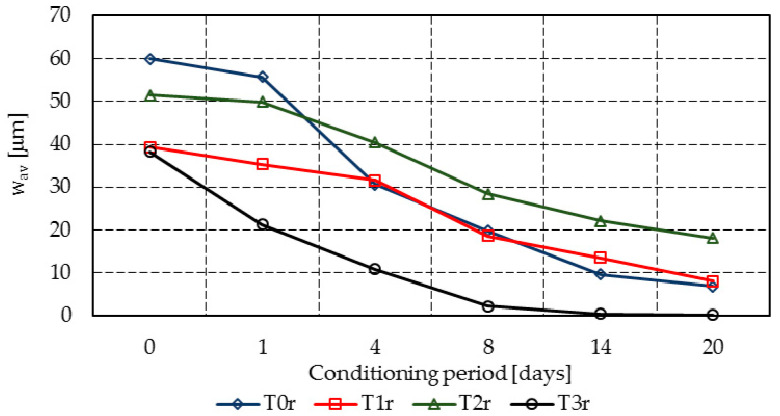
Mean crack progress in mixtures T0r–T3r.

**Figure 5:**
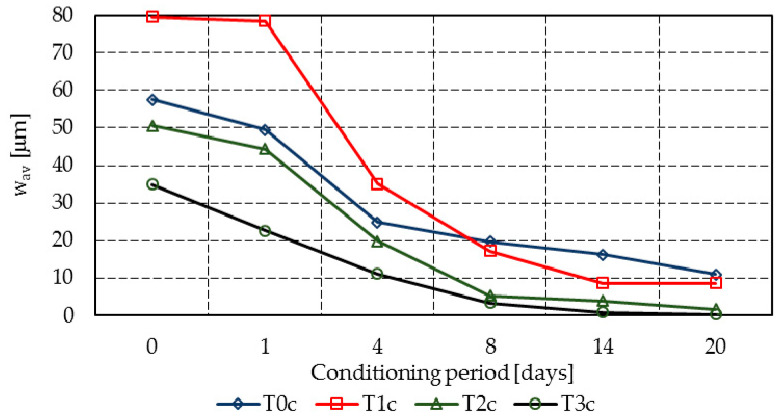
Mean crack progress in mixtures T0c–T3c.

Table 4 presents the regression of the individual crack widths registered on all specimens, while Figure 4 and Figure 5 summarize graphically the width progress of the mean cracks in all mixtures.

Despite the initial predictions, the maximum aggregate size was not found to influence the initial cracking behavior. While mixture T0r registered a crack width similar to T0c (i.e., just 2.4 µm larger), the same tendency was also found in the rest of the mixes, except mixes T1r and T1c, where the results are significantly different.

**Figure 6:**
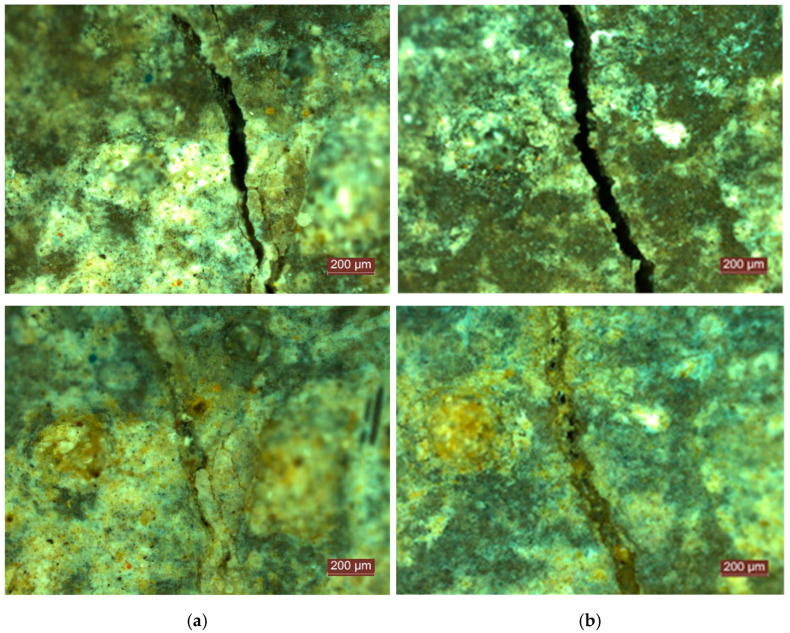
Initial crack and self-sealing products at 20 days of the reference mixes: (**a**) T0r; (**b**) T0c.

**Figure 7:**
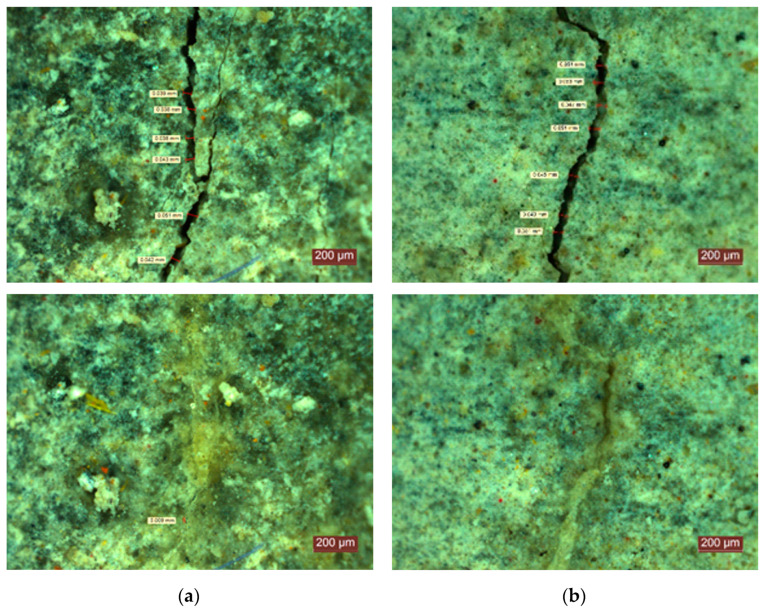
Initial crack and self-sealing products at 20 days for an ICW content of 3%: (**a**) T3r; (**b**) T3c.

Mixtures T3r and T3c entirely or almost entirely filled the cracks within 14 days, at which point the registered closure rates were 98.9% and 97.3%, respectively. However, at 20 days, T3r reached 99.6% and T3c reached 99.3%, while the reference mixes T0r and T0c presented closure rates of 88.6% and 80.8%, respectively (see Figure 6 compared to Figure 7).

Mixture T2c achieved a similar performance, with a crack closure of 96.7% at 20 days. Mixtures T1c and T1r had similar tendencies (i.e., crack closures of 79.5% and 89.2%, respectively, at 20 days), while the results for T2r were divergent (i.e., just 64.7% at 20 days).

**Figure 8:**
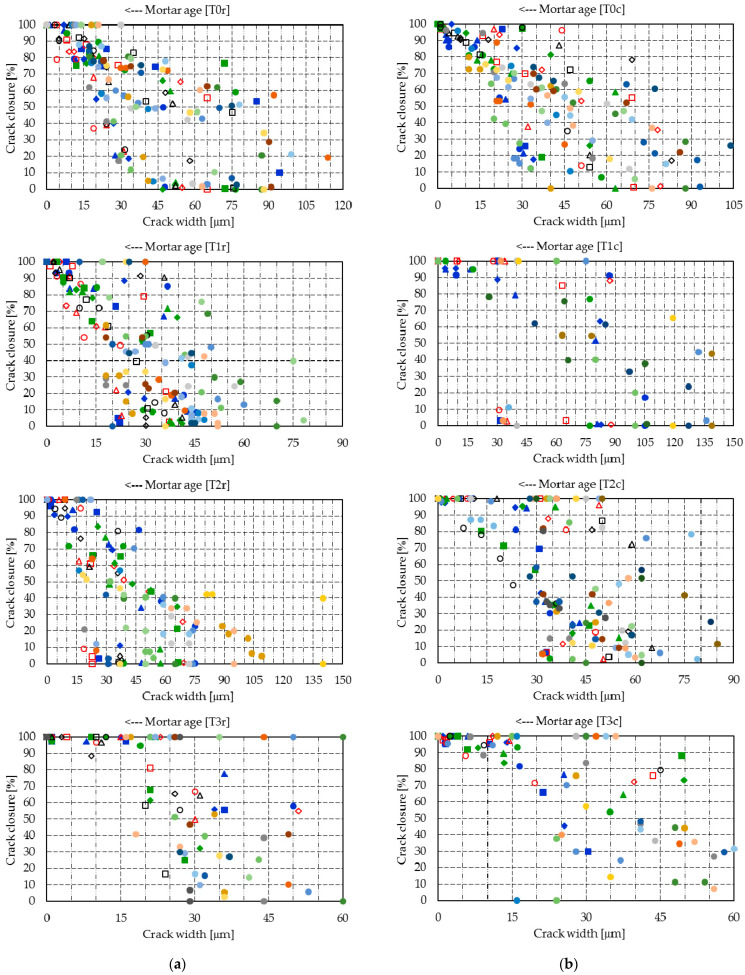
Segmented crack closures—markers correspond to the readings of each crack segment: (**a**) T0r–T3r; (**b**) T0c–T3c.

**Figure 9:**
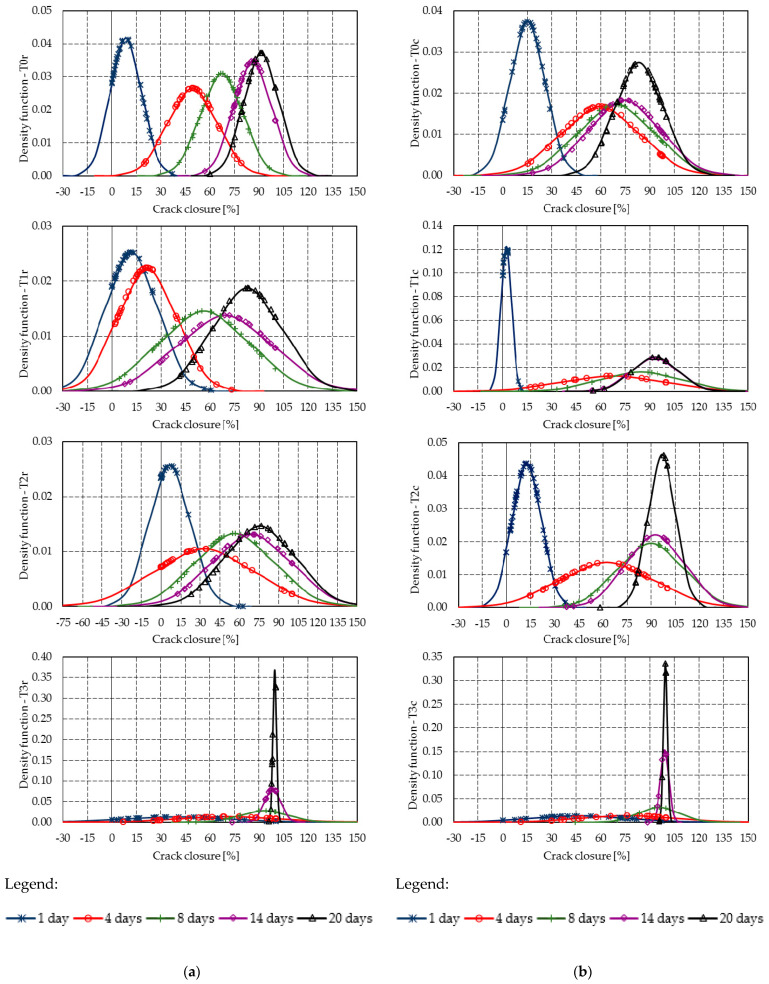
Gaussian distributions of the crack closure: (**a**) T0r–T3r; (**b**) T0c–T3c.

Figure 8 presents the raw segmented data for the crack closure and Figure 9 shows the Gaussian distribution registered for the raw segmented data. Considering the physical significance of the crack closure is described as a value between 0% and 100%, Table 5 presents the mean crack closure and standard deviation at 20 days, and the probabilities of reaching several different intervals of crack closure. In probabilistic terms, the 3% ICW powder showed the best sealing capacity for both mixture types. At 20 days, T3r showed a probability of 0.36 of full closure, and a probability of 0.98 of at least 75% closure, while T3c had a full closure probability of 0.35, and a probability of 0.98 for a closure of more than 75%. A mention should be made of the reduction in the standard deviation, which dropped spectacularly at 20 days with the ICW content of 3%. T2c, with an ICW content of 2%, presented a similar closure trend.

The results clearly show that, in structural mortars made with Portland cement and fly ash in similar proportions, ICW contributes to the sealing of crack widths of up to 100 µm within 20 days. Sisomphon et al. [22] reported the sealing of cracks up to 400 µm at 28 days in mortars made with Portland cement. Research performed by Roig-Flores et al. [41] and Ferrara et. al [39] confirms these results. Jaroenratanapirom and Sahamitmongkol [53] investigated mortars containing various additional cementitious materials, such as fly ash. They concluded that all mortars show a self-healing capacity to some extent. For crack widths up to 50 μm, ICW showed the best self-healing capacity. Chandra et. al. [54] found that a 30% fly ash content and a 1.5% ICW content achieve 102% mechanical strength recovery under water immersion conditions. After water immersion, wet–dry cycles ensure the best self-healing performance. Wang et al. [55] concluded recently that the excessive use of fly ash has a negative impact upon the mortar’s self-healing potential if the content is over 10 wt. %. However, the contributions of both fly ash and ICW are considerably increased by the presence of water. When ICW is added to cementitious materials, the main healing product is CaCO_3_. The sealing closure is related to the concentration of Ca^2+^, CO_2_, and water at the crack entrance. The chemical reactions are:

(6) $${\mathrm{CO}}_{2}+H_{2}O↔H^{+}+{\mathrm{HCO}}^{3-}↔H^{+}+{\mathrm{CO}}_{3}^{2-}$$

(7) $${\mathrm{CO}}_{3}^{2-}+{\mathrm{CO}}^{2+}\to {\mathrm{CaCO}}_{3}$$

Even if the fly ash content does not affect the crystallization reaction of ICW, it generates C–S–H gel and helps with crack self-sealing via the reaction:

(8) $$\left(0.8\sim 1.5\right)\mathrm{Ca}\left({\mathrm{OH}}_{2}\right)+{\mathrm{SiOH}}_{2}+\left|n-\left(0.8\sim 1.5\right)\right|H_{2}O\to \left(0.8\sim 1.5\right)\mathrm{CaO}\cdot {\mathrm{SiO}}_{2}\cdot nH_{2}O$$

### 3.2. Self-Sealing Closure Rate

**Figure 10:**
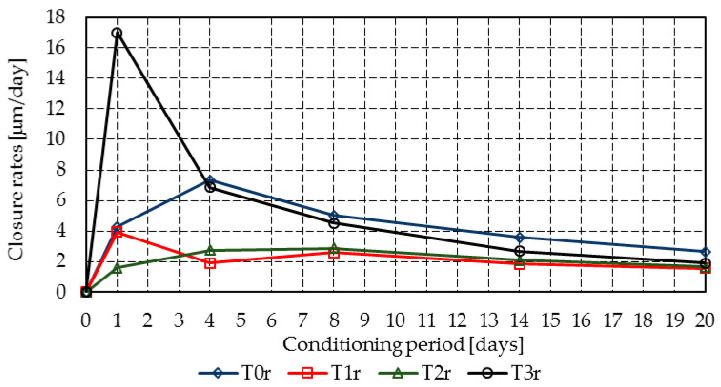
Mean closure rates in mixtures T0r–T3r.

**Figure 11:**
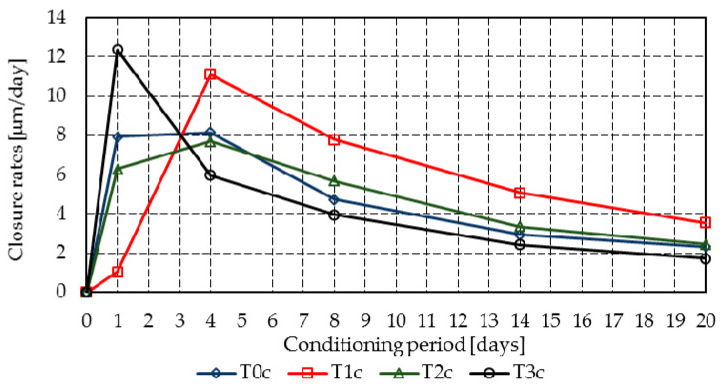
Mean closure rates in mixtures T0c–T3c.

**Figure 12:**
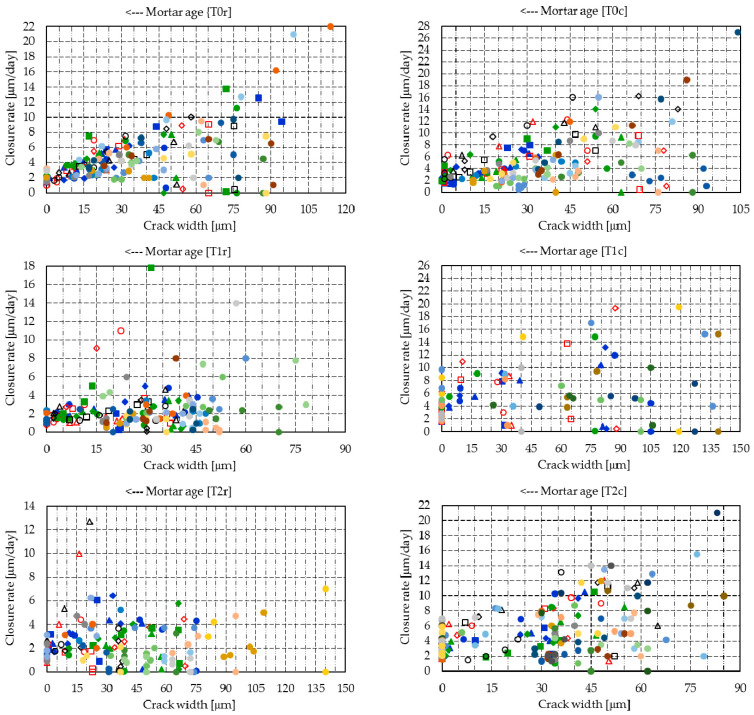

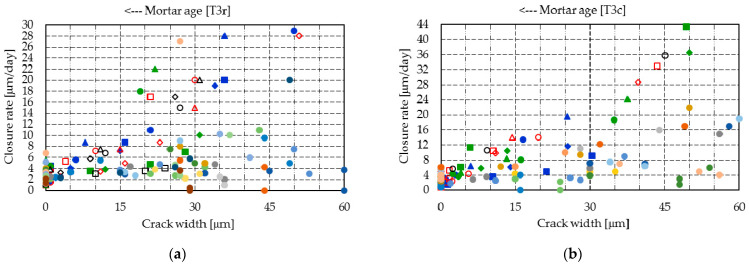
Segmented closure rates—markers correspond to the readings of each crack segment: (**a**) T0r–T3r; (**b**) T0c–T3c.

**Figure 13:**
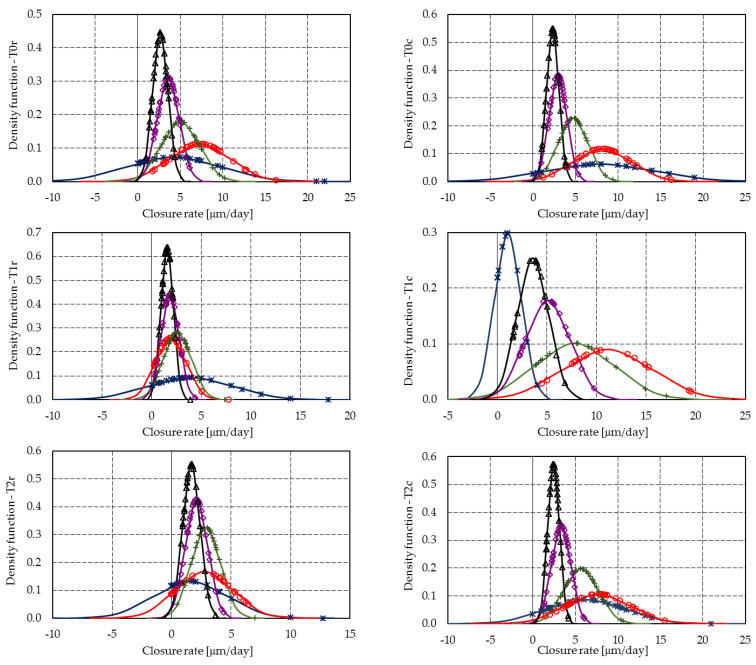

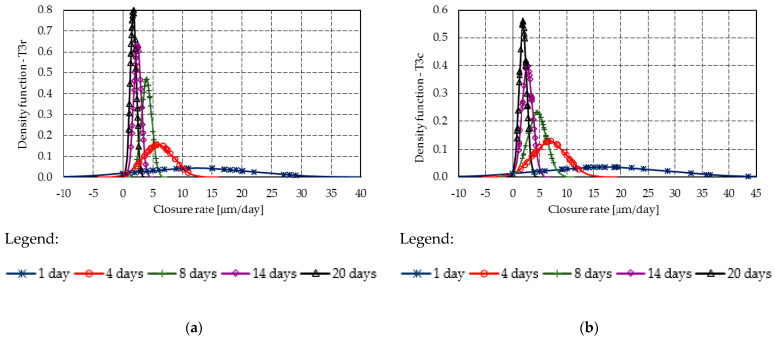
Gaussian distributions of the closure rate: (**a**) T0r–T3r; (**b**) T0c–T3c.

Figure 10 and Figure 11 summarize the crack closure rate of the individual cracks, Figure 12 presents the raw segmented data for the crack closure, and Figure 13 shows the Gaussian distributions of the closure rate. 

Mixtures T3r and T3c achieved a high initial closure rate of 12.4 µm/day and 17.0 µm/day, all other mixtures having closure rates below 8.0 µm/day, with the exception of mixture T1c, which reached 11.1 µm/day on the fourth day. All mixtures presented a gradual reduction in the mean closure rate up to 20 days. However, due to the delayed hydration of fly ash, further increases may occur.

Because the results of the probabilistic analysis are strongly influenced by the closure of many crack segments, the analysis will be made on mortar at 8 days of age. Table 6 shows the probabilistic parameters and the probabilities of various significant closure rates at 8 days. 

At 8 days, despite the fact that mixtures T1c and T2c display the highest likelihood of achieving closure rates above 6 μm/day (i.e., at least 120 μm crack closure in 20 days), for closure rates above 3 μm/day (i.e., at least 60 μm crack closure in 20 days), mixes T0r and T0c–T3c achieve similar probabilities, which vary between 0.78 and 0.91. It must be noted that, within 1 day, T3r and T3c developed the highest closure rates.

## 4. Conclusions

Here, the influence of ICW content (i.e., 1–3% of Portland cement mass) upon early-age crack sealing in structural mortar has been investigated. The experimental evidence clearly shows that an ICW content of 3% achieves the best crack closure ability. However, these values are also influenced by the mechanical properties that govern the initial crack’s width, the fracturing mode, and most importantly, the exposure conditions. In probabilistic terms, all the analyzed mixtures showed a significant ability to fill cracks with widths of up to 90–180 μm at early ages of up to a month.

The autogenous early-age self-sealing potential displayed by mortar mixtures with a maximum grain size below 4 mm is clear, and opens the way for larger well-graded aggregates with adequate self-sealing/self-healing abilities, which can also achieve superior time-dependent mechanical performances.

## Figures and Tables

**Table 1:** Reference mix contents T0r and T0c.

Mixture	Cement (kg)	Fly Ash (kg)	Sand/Gravel (kg)	MS (kg)	HRWRA (kg)	Water (kg)	PVA (kg)
T0r	580	650	476	141	12.75	327	26
T0c	580	650	476	141	12.75	327	26

Note: Cement (density: 1.07 g/cm^3^), fly ash (density: 0.89 g/cm^3^), standard sand (density: 1.65 g/cm^3^), fine gravel (density: 1.55 g/cm^3^), MS (density: 1.19 g/cm^3^), HRWRA (density: 0.77 g/cm^3^), water (density: 0.99 g/cm^3^), PVA (density: 1.30 g/cm^3^). Values were determined with a standard deviation of ±1%.

**Table 2:** Relative mass mix proportions.

Mixture	Cement	Fly Ash	Sand/Gravel	MS	HRWRA	Water	PVA	ICW
T0r ^1^, T0c ^2^	1	1.12	0.82	0.24	0.02	0.56	0.04	-
T1r ^1^, T1c ^2^		1.11						0.01
T2r ^1^, T2c ^2^		1.10						0.02
T3tr ^1^, T3c ^2^		1.09						0.03

^1^ Specimens made with standard quartz sand; ^2^ Comparative specimens made with quartz gravel.

**Table 3:** Modulus of rupture for the tested specimens and mixtures.

Mixture	Density (kg/m^3^)	Effective Flexural Strength (MPa)	Mean Flexural Strength (MPa)
T0r	1921	17.2/16.7/16.3	16.7
T1r	1923	16.9/15.1/15.2	15.7
T2r	1927	18.2/20.1/18.5	18.9
T3r	1931	19.3/21.5/19.8	20.2
T0c	1918	15.9/16.4/15.0	15.8
T1c	1922	15.3/16.2/17.2	16.2
T2c	1923	16.7/17.3/14.9	16.3
T2c	1925	16.7/16.1/16.7	16.5

**Table 4:** Crack progress in the specimens and mean crack widths for mixtures.

Mixture	Crack Widths (mm) in the Conditioning Period					
	0 Days	1 Day	4 Days	8 Days	14 Days	20 Days
T0r	53.9/65.5/60.4	50.2/60.7/56.1	23.2/37.6/31.1	14.2/25.0/20.1	6.8/12.4/9.9	4.4/9.0/7.0
	59.9 ± 5.8	55.7 ± 5.3	30.6 ± 7.2	19.8 ± 5.4	9.7 ± 2.8	6.8 ± 2.3
T1r	37.2/46.3/34.5	32.5/42.6/31.2	29.4/37.8/27.8	9.3/29.2/17.4	4.7/23.0/12.9	3.2/13.4/7.7
	39.3 ± 6.2	35.4 ± 6.2	31.6 ± 5.3	18.6 ± 10.0	13.5 ± 9.2	8.1 ± 5.1
T2r	53.2/59.1/42.0	50.5/58.1/40.9	36.4/50.8/34.2	21.4/38.9/24.9	13.1/33.0/20.1	8.9/28.6/16.9
	51.4 ± 8.7	49.8 ± 8.6	40.5 ± 9.0	28.4 ± 9.3	22.1 ± 10.1	18.1 ± 9.9
T3r	32.6/39.0/33.5	13.6/32.3/22.0	4.3/18.3/11.0	1.3/5.6/3.3	0.5/1.4/0.9	0.2/0.3/0.2
	35.0 ± 3.5	22.6 ± 9.4	11.2 ± 7.0	3.4 ± 2.1	0.9 ± 0.4	0.3 ± 0.1
T0c	49.3/61.5/61.7	41.9/53.6/ 53.2	13.2/34.4/27.0	8.0/29.6/21.4	5.7/25.4/17.7	4.6/17.8/10.8
	57.5 ± 7.1	49.6 ± 6.6	24.9 ± 10.8	19.7 ± 10.9	16.3 ± 9.9	11.0 ± 6.6
T1c	73.5/88.2/76.9	72.6/87.2/75.7	27.2/46.1/31.9	10.2/28.4/12.9	8.0/14.6/3.3	7.8/14.0/3.0
	79.5 ± 7.7	78.5 ± 7.7	35.1 ± 9.8	17.3 ± 10.0	8.6 ± 5.7	8.3 ± 5.5
T2c	48.2/54.5/49.3	41.7/48.2/43.3	14.9/25.9/18.9	2.3/8.1/5.7	1.3/6.3/4.2	0.9/2.4/1.8
	50.7 ± 3.4	44.4 ± 3.4	19.9 ± 5.6	5.3 ± 2.9	3.9 ± 2.5	1.7 ± 0.7
T3c	33.2/39.0/42.6	10.0/29.4/24.6	2.8/17.0/12.8	0.5/3.5/2.6	0.2/0.6/0.5	0.2/0.1/0.2
	38.3 ± 4.8	21.3 ± 10.1	10.9 ± 7.3	2.2 ± 1.5	0.4 ± 0.2	0.2 ± 0.0

**Table 5:** Probabilistic parameters and probabilities of crack closure at 20 days.

Mixture	μ/σ (%)	Probabilities for Significant Crack Closure Intervals of		
		≥50% and <75%	≥75% and <100%	100%
T0r	91.3/10.7	0.07	0.59	0.21
T1r	82.7/21.2	0.20	0.43	0.21
T2r	75.7/27.2	0.33	0.32	0.19
T3r	99.5/1.0	0.00	0.62	0.36
T0c	83.4/14.6	0.17	0.69	0.13
T1c	93.2/13.7	0.15	0.53	0.31
T2c	96.7/8.6	0.02	0.64	0.35
T3c	96.7/10.7	0.00	0.63	0.35

**Table 6:** Probabilistic parameters and probabilities of closure rates at 8 days.

Mixture	μ/σ [%]	Probability of Closure Rates		
		≥1 μm/day	≥3 μm/day	≥6.0 μm/day
T0r	5.02/2.22	0.97	0.78	0.35
T1r	1.56/0.63	0.87	0.38	0.01
T2r	2.88/1.22	0.94	0.45	0.01
T3r	3.95/0.50	0.99	0.87	0.01
T0c	4.73/1.74	1.00	0.78	0.26
T1c	7.78/3.94	0.96	0.89	0.68
T2c	5.67/2.02	1.00	0.91	0.42
T3c	1.91/0.71	1.00	0.81	0.19

## Data Availability

The data presented in this study are available on request from the authors.
